# Meet a Chemist Repurposing Tech from the 1850s to Fight Water Scarcity

**DOI:** 10.1021/acscentsci.6c00474

**Published:** 2026-04-07

**Authors:** Diana Kruzman

## Abstract

Juan Felipe Torres’s new spin on an old technique aims to desalinate water at scale.

## Graphical abstract

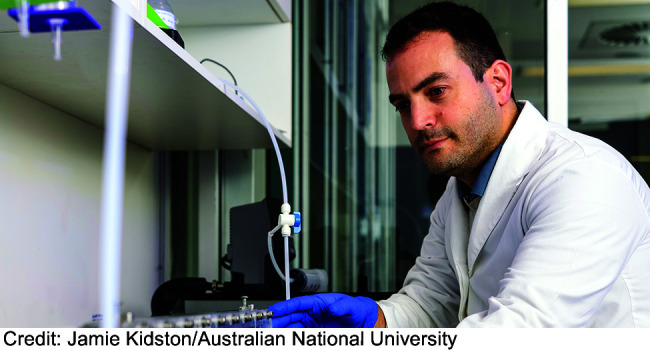

As climate change drives hotter, drier weather and a population boom strains the limited water supply in arid regions, a growing number of cities and countries are turning to desalination to meet their freshwater needs. The global demand for desalinated seawater is expected to double by 2030. More than 21,000 desalination plants are currently in operation, up from 14,000 in 2008.

**Figure:**
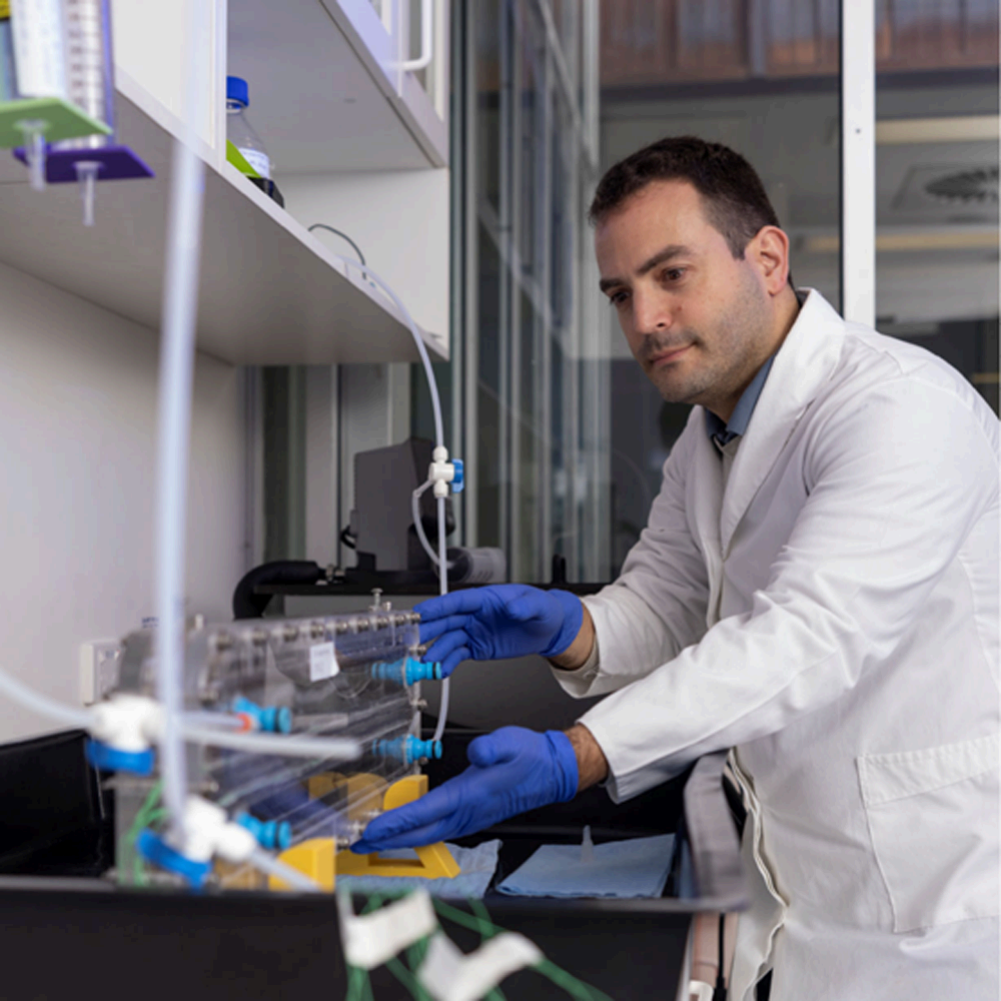
Juan Felipe Torres stands with his thermodiffusion device. Torres developed his technique for using thermodiffusion to desalinate seawater after helping to clean contaminated water from the Fukushima nuclear meltdown. Credit: Jamie Kidston/Australian National University.

But the process that powers most of these plants, called reverse osmosis, is energy intensive and can be damaging to the marine environment. It requires large amounts of electricity (most often supplied by burning fossil fuels) to push water through a membrane and separate salt from H_2_O molecules, producing a heavy saline brine as a byproduct that’s released back into the ocean.

Juan Felipe Torres, a researcher at the Australian National University, has developed a technique for desalinating seawater that he believes can address all these issues at once. It’s based on a method called thermodiffusion, which has been known to science since the 1850s but operates too slowly to be practical for most real-world applications. With Torres’s engineering tweaks, the method is much faster. He’s now working on patenting it and spreading it to various industries through his company, Soret Technologies.

Diana Kruzman spoke with Torres about how thermodiffusion could outcompete reverse osmosis in certain cases and how it can be applied to lithium mining and chemical engineering. This interview was edited for length and clarity.

## What’s the current state of desalination technology?

The industry worldwide right now is relying on a very old technology. It’s called reverse osmosis, and the first commercial plant in the world opened in California 60 years ago. Fast-forward 60 years, and you would think this technology took over and solved our problems. But in reality, we’ve satisfied less than 0.5% of our freshwater needs by desalination, and more than a billion people worldwide are suffering from water scarcity. So the technology that we’re using now is old, and it does not meet the needs we have.

Although the energy efficiency of reverse osmosis is outstanding, there are risks of long-term use—membranes degrade, and they’re difficult to maintain. And there’s also the brine problem. The recovery rate, which is how much water we can purify from seawater for a given technology, for reverse osmosis is 50%. That means that for every cubic meter of fresh water that is produced, a cubic meter of brine is produced as well. And that brine is hypersaline, double the salt concentration [of seawater]. And when it’s dumped into the ocean, it sinks to the seabed and has a big environmental impact.

## What exactly is thermodiffusion?

Thermodiffusion is the transport of species, either ions or dissolved solids, when there’s a temperature gradient. So one side is hot, the other side is cold, and those species would move either to the hot or to the cold. And sodium chloride ions, in the case of seawater, move spontaneously to the cold side.

**Figure:**
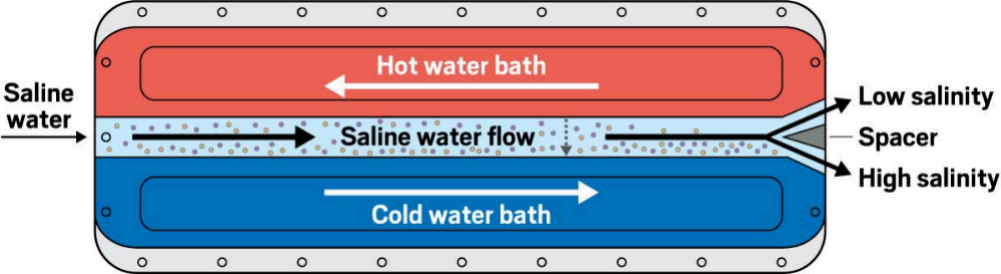
**How thermodiffusion desalinates water**. Thermodiffusion works by leveraging the natural properties of sodium chloride ions, which move spontaneously toward the cold side of a channel (shown). In Torres’s device, multiple channels are combined to enhance thermodiffusive separation. Credit: Yang H. Ku/C&EN.

## How did you start working on it?

I started working at Toshiba a couple of years after the Fukushima [Daiichi Nuclear Power Plant] disaster. At the time, Toshiba was in charge of cleaning all this contaminated water [from the nuclear meltdown]. And while doing research, I found that thermodiffusion was used in the Manhattan Project to enrich uranium; the first bomb was actually enriched halfway through thermodiffusion. This background, and the need to clean and purify water that had this nuclear waste content, led me to study a bit more about the potential of thermodiffusion as a separation technology.

## Why haven’t we used thermodiffusion for desalination before?

Thermodiffusion has never been used for desalination because it’s a weak phenomenon. It takes a long time to produce small concentration differences. So this is where the engineering challenge is. How can we enhance thermodiffusive separation while keeping recovery rates of 50% or more, and without compromising energy efficiency?

This is where our invention comes into play. It’s called multichannel thermodiffusion, which is a network of channels that enhance thermodiffusive separation. We arrange them in a cascaded structure that can be 3D printed or produced en masse using injection molding.

Water flows through a rectangular channel where the top wall is hot and the bottom wall is cold. The ions move spontaneously to the cold bottom wall. Once the flow reaches the end of the channel, the high-concentration [stream] goes to the right, and the low-concentration [stream] moves to the left. We use neighboring channels to reconnect. . . low-concentration streams [which we then run through thermodiffusive separation again]. So at the end, we’re able to enhance the separation substantially.

## Can your method handle desalination on the massive scales needed to aid the water crisis?

It’s very scalable using conventional manufacturing processes and conventional materials. So we don’t need fancy manufacturing or fancy chemical synthesis or anything like that. We need injection molding, which is how we build Legos; millions and millions of Lego pieces, with injection molding, are very low cost. And we need conventional materials such as stainless steel, something that doesn’t corrode too much when it’s in contact with seawater.

## What kind of power difference is there between current reverse osmosis and your new technique?

Large-scale reverse osmosis uses between 3 and 5 kW·h to produce 1 m^3^ of water. The consumption of our technology is around 300 kW·h/m^3^. But not all energy is made equal.

Reverse osmosis is driven by pressure differences produced by very energy-intensive pumps. They use a lot of electric power.

Our process uses heat to drive the separation, and the interesting thing is that this heat is a low-temperature heat, below 100 °C. You can use the heat from data centers, for example, [or] moderate- to low-temperature industrial processes.

## What kind of impact do you expect thermodiffusion to have?

One of the key things we’re looking at is to be a mitigating technology for climate change. Large population centers, like Johannesburg or Mexico City, have been on the news frequently due to the risk of “day zero”: the time when the water is just going to run out.

[I see potential for a] hybrid reverse osmosis–thermodiffusive desalination system, where thermodiffusive desalination treats the brine to increase the recovery rate [and bring it] close to 100%. [Note that this process is known as zero liquid discharge, a type of desalination where only solid waste is produced and all the water is recovered.] You’ll be able to have a reliable supply of fresh water to sustain these large population centers, which is absolutely critical in this time of climate change.

## What other applications does this technique have? I read about lithium extraction, for example.

At the industry level, we would like to replace two technologies. One is evaporation ponds—big pools of seawater or lithium brines, and we wait for months and months for them to dry out. So this is a very slow process [that] consumes huge amounts of land. We found that our technology actually is cheaper than evaporation ponds. So there’s a massive opportunity for resource recovery and growing mining with our technology. We’re exploring lithium bids; we’re exploring potash, which is a fertilizer.

From a chemical engineering perspective, we’re looking at concentrating strong acids and strong bases. There’s a strong base called sodium hydroxide or caustic soda. We’ve shown that multichannel thermodiffusion can concentrate caustic soda to 50%, which is widely used in industry. So as a fluid-refining technology, it will have tremendous implications in the chemical engineering field.

*Diana Kruzman is a freelance contributor to* Chemical & Engineering News, *an independent news publication of the American Chemical Society.*

